# Seasonal swarming behavior of *Myotis* bats revealed by integrated monitoring, involving passive acoustic monitoring with automated analysis, trapping, and video monitoring

**DOI:** 10.1002/ece3.9344

**Published:** 2022-09-23

**Authors:** Robert J. Thomas, Stephen P. Davison

**Affiliations:** ^1^ Cardiff University Cardiff UK; ^2^ Eco‐explore Community Interest Company Cardiff UK; ^3^ Independent Researcher Newport UK

**Keywords:** acoustic monitoring, bats, myotis, swarming

## Abstract

Bat abundance, diversity, and behavior can be monitored by capturing bats for identification and measurement in the hand, but this has several disadvantages. These include disturbance to the bats, which limits the frequency with which captures can be made at an individual capture site, and potentially alters the behaviors being studied. Infrared video monitoring, passive acoustic recording and automated analysis and identification of bat calls offers an alternative set of noninvasive methods for monitoring bats. In this study, we examine the effectiveness of acoustic monitoring in comparison with capture‐based and video monitoring of seasonal swarming behavior among several species of *Myotis* bats in southern Britain. We applied these complementary approaches to describe seasonal, overnight, and species‐specific variation in swarming behavior in a multispecies community of *Myotis* bats. We show that the three monitoring approaches have advantages and disadvantages for different tasks, but can be viewed as highly complementary methods for addressing different types of research questions. In our study of swarming behavior, capture and examination of bats in the hand was necessary for measuring sex ratios, reproductive status, and even for confirmation of species identification for some difficult to separate taxa. Capture is also an essential aspect of tagging bats for individual identification and tracking studies. Video monitoring is useful for understanding the behavior of bats at swarming sites, and measuring the flux of individuals into and out of roosting or swarming sites. Passive acoustic monitoring is a valuable noninvasive method for continuous monitoring of within‐night, seasonal, and between‐year variation in the abundance of bat calls. These can be used as an index of variation in relative abundance within—but not between—bat species.

## Graphical abstract

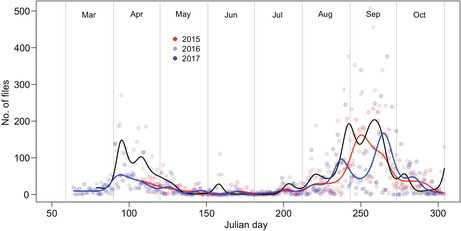

## 1 INTRODUCTION

Monitoring of animal populations is important for identifying changes in species diversity, population size and demographics, for locating habitats and locations on which those populations rely, and for identifying methods to mitigate population declines (Barlow et al., 2015; JNCC, 2019; Mathews et al., 2020; National Bat Monitoring Programme Annual Report, 2021; Tuneu‐Corrala et al., 2020). However, such monitoring is difficult for nocturnal taxa such as bats, due to the difficulty of observing, identifying, and counting them directly. In this study, we examine and compare the information provided by three complementary bat‐monitoring methods; (i) acoustic monitoring combined with automated identification, (ii) trapping, and (iii) video monitoring. We apply these three complementary methods to investigate the poorly understood phenomenon of seasonal swarming behavior, among several species of *Myotis* bats in southern Britain.

Bats make species‐specific calls for echolocation and social functions, enabling the presence of different species and changes in their calling activity to be monitored noninvasively by passive recording and identification of their calls (Fenton, 2003), and allow estimation of activity (Gibb et al., 2019; Marques et al., 2013) Bats do, however, vary their calls in relation to different habitats, functions, and the presence of other bats; this can result in difficulties of species recognition where the calls of one species are similar to the calls of another (Barataud, 2015). As a result, for some species, capture and examination in the hand (or even DNA confirmation) may be needed to identify individual bats to species level (Kuenzi & Morrison, 1998). Furthermore, different bat species emit different intensity or directionality of calls, and at different frequencies (Anderson & Racey, 1991; Barataud, 2015; Goerlitz et al., 2010), with the inevitable result that some species' ultrasonic calls are easier to detect than others, leading to a bias in the species abundance being detected using acoustic methods. In spite of these issues, recording of acoustic activity can give a good indication of activity patterns (Beason et al., 2020).

Trapping of bats using mist nets or harp traps is an important monitoring method, complimentary to acoustic monitoring. Capture allows close examination in the hand, facilitating visual identification based on morphological traits, and sampling of genetic material. Like acoustic monitoring, trapping has inherent biases, however, as some species of bats are better at either avoiding traps or escaping from them than others (MacCarthy et al., 2006; Robbins et al., 2008). Bat species also differ in the height at which they fly above the ground, and therefore, higher‐flying species are less likely to be captured in traps, which are typically set 1–4 m above ground level. As a result, trapping of bats is not necessarily a good measure of the absolute numbers or number of species present (MacSwiney et al., 2008). For example, Leon‐Tapia and Hortelano‐Moncada (2016) reported that in their study in Mexico, 12 species of bats were detected by using ultrasonic detectors, whereas only five species were trapped.

Furthermore, trapping inevitably disturbs a bat's natural activity and hence can only be undertaken infrequently. Indeed, trapping on successive nights leads to reduced catches, indicating some alteration in behavior (Kunz & Brock, 1975). Thus, this method cannot be utilized to describe variation in activity over short time periods (e.g., within or between successive nights), even were all species to be trapped. Likewise, infrared video monitoring can be very effective in quantifying bat activity (Brown & Scroggie, 2008) and is not believed to disturb bats, but rarely allows identification to species level where cryptic species are involved (personal observation). Given the limitations of each of these different approaches, a combination of methods is needed to characterize local bat assemblages and changes in bat activity over time (Grandison, 2004; Kunz et al., 2009).

In Europe, species of bats within the *Myotis* genus (including Brandt's bats *Myotis brandtii*, Daubenton's bats *Myotis daubentonii*, Natterer's bats *Myotis nattereri* and whiskered bats *Myotis mystacinus*), as well as other species such as the barbastelle bat *Barbastella barbastellus* and the brown long‐eared bat *Plecotus auritus*, aggregate at certain sites in Autumn, in a poorly understood social activity known as “Autumn swarming” (Fenton, 1969; Glover & Altringham, 2008; Parsons, Jones, & Greenaway, 2003; Parsons, Jones, Davidson‐Watts, et al., 2003; Rivers et al., 2006). The nature of swarming and its exact functions remain poorly understood; in his seminal paper on swarming of bats, Fenton (1969) refers to two phases of swarming. He suggested that the earlier phase was to familiarize juveniles with potential hibernacula, whereas the latter phase was concerned with fat deposition and mating. These results were gained from 49 nights of trapping in August and September over a three‐year period at 10 caves. While this was a considerable sampling effort, it may have missed differences (if any) between the sites and the species due to the relatively infrequent sampling at each site in each year. More recent studies suggest that in swarming aggregations, bats appear to congregate primarily for the purpose of mating. Sex ratios at swarming sites on any specific night are heavily biased toward males, with females attend swarming sites sporadically, probably in order to mate (Furmankiewicz et al., 2013; Glover & Altringham, 2008; Parsons, Jones, & Greenaway, 2003; Parsons, Jones, Davidson‐Watts, et al., 2003; Rivers et al., 2006; van Shaik et al., 2015). Bats may attend swarming aggregations from a wide geographical area; for example, Rivers et al. (2006) found that a swarming site for Natterer's bats had a catchment radius of up to 60 km. Until now, little is known about changes in activity throughout the swarming period, including Fenton's (1969) suggestion of two swarming phases. Likewise, little is known about species differences in patterns of swarming, and how these may be affected by environmental conditions.

Previous studies of Autumn swarming in these species within the British Isles have usually focused on underground formations (Glover & Altringham, 2008; Parsons, Jones, Davidson‐Watts, et al., 2003), with common features including a well‐developed underground chamber, absence of water in the chamber, and shelter (e.g., vegetation) at the entrance. There is no correlation between swarming activity and the size of entrance opening (Glover & Altringham, 2008). Parsons, Jones, and Greenaway (2003) studying swarming sites in the Yorkshire Dales, NE England, reported that peak autumn swarming activity generally occurs 6–7 h after sunset, is reduced by rain, and is positively correlated with ambient temperature. The diel timing of swarming may vary in response to changes between years, for example due to differences in weather conditions; for example, Glover and Altringham (2008) found that the peak of autumn swarming activity during the night at caves in Yorkshire occurred 3–4 h after dusk—earlier in the night than reported by Parsons, Jones, and Greenaway (2003). The swarming season for *Myotis* bats is late summer to early autumn, with Brandt's bats and Daubenton's bats swarming relatively early in this period, while Natterer's bats and whiskered bats swarm later in the season (Parsons, Jones, & Greenaway, 2003).

Monitoring of bats at swarming sites is of particular importance, given the large numbers of individuals involved, the large distances that bats travel to attend swarming sites, and the likely role of swarming in the reproductive behavior of bats. van Shaik et al. (2015) report that bats hibernate where they swarm, enabling swarming sites to be used as indicators of hibernation sites, which is particularly useful where the bats hibernate in inaccessible crevices where they cannot be directly observed and counted. Systematic monitoring of swarming within and between years is logistically challenging, however. Studies of swarming bats typically involve capture of bats, with the limitations and biases described above. Furthermore, although individual adult males can stay at the swarming site for many nights, turnover of individuals between nights and potential trap‐shyness effects lead to recapture rates of males being very low (personal observations). In addition to trapping and handling, swarming activity can be monitored much less intrusively using passive logging of bat ultrasonic calls. Glover and Altringham (2008) and Parsons, Jones, and Greenaway (2003) report that there is a strong positive correlation between the number of bat echo location calls logged at swarming sites, and the numbers of bats caught. Records of bat activity in these previous studies only comprised the total number of bat passes (sequences of calls recorded by the bat detector), with no attempt to identify individual species of bat.

In this study, the “Bat Classify” software (Scott & Altringham, 2014) was used to investigate *Myotis* bat activity at a cave‐entrance swarming site across the autumn swarming period, to determine whether the activity patterns of different species as measured by the classification software is consistent with the activity patterns as measured by trapping. The continual automated acoustic monitoring of bats across the active season provides much more detailed temporal information on bat activity than sporadic trapping sessions can. Infrared video monitoring was also used to visualize the behavior of swarming bats at the swarming site, and to quantify the movement of bats into and out of the cave over the course of multiple nights, which is not revealed by acoustic or manual trapping methods. These combined methods were used to examine (a) seasonal and (b) overnight patterns of activity of each *Myotis* species present at a multispecies swarming site in the Wye Valley, on the border between Wales and England. These temporal patterns of activity were (c) compared to examine differences between species in swarming behavior.

## 2 MATERIALS AND METHODS

### 2.1 Study area

The study was carried out in an area broadleaved woodland to the North of Chepstow (Wales, UK; centered on latitude 51.6711, longitude −2.6862) on the steep sides of the River Wye valley. In this area are several natural cave formations and man‐made openings in the predominantly limestone rock strata. One of these caves within the woodland (at latitude 51.67216, longitude −2.68493) has a small opening (~600 × 500 mm aperture) but extends a considerable distance downward (at least 50 m). This cave, referred to as “Middle Earth,” has recently been found to be a site used for swarming by multiple species of bats, including Lesser Horseshoe Bats *Rhinolophus hipposideros* (Davison & Thomas, 2017) and several Myotis species, as shown below. In addition to the main data collection at “Middle Earth” cave during 2017 and 2018, additional monitoring was carried out at the nearby “Hobbit Hole” cave (51.6707, −2.6864, described in Davison & Thomas, 2017) within the same woodland, during 2015–2017 (see below).

### 2.2 Measurement of bat activity

#### 2.2.1 Acoustic monitoring

A Titley Scientific Anabat Swift bat detector (https://www.titley‐scientific.com) was deployed to monitor bat calling activity immediately outside the cave. It was set to record full spectrum echo location calls, at a 500 kHz sampling frequency. The detector switched on automatically at 15 min before sunset and turned off 15 min after sunrise. Monitoring in 2017 was undertaken from 26 July to 29 October, with 14 of the 96 nights within this period being missed due to the equipment failing to activate. Monitoring in 2018 was undertaken from 23 March until 2 November; no nights of data capture were missed. In addition, a Titley Scientific Walkabout handheld bat detector (https://www.titley‐scientific.com) was used occasionally in the nearby woodland to detect bat activity in the wider area, recording at 500 kHz in full spectrum.

Manual identification of echo location calls was undertaken by visual inspection of sonograms using “Anabat Insight” software (https://www.titley‐scientific.com) by one observer (SPD). These manual identifications were compared with the results of automated analysis of calls, undertaken using “Bat Classify” software (https://bitbucket.org/chrisscott/batclassify/downloads). This software was chosen as it is claimed that it reliably identifies the calls of most British woodland bats, including all Myotis species in the study area (Scott & Altringham, 2014). This automated software does not attempt to differentiate between calls of whiskered and Brandt's bats, due to the similarity between the echo location calls of these two species. Only calls that the Bat Classify software identified to species level with 80% confidence levels or more were used for the purpose of this study. The focus of this study was not to compare different software options, but to test the effectiveness of Bat Classify software against manual capture methods.

In parallel with, and before this study, 3 years of bat echolocation data (2015–2017) were collected at the nearby cave “Hobbit Hole” (see Section 2.1) and analyzed using a Wildlife Acoustics SM3 Bat detector recording in “zero‐crossing” mode (see Davison & Thomas, 2017). Unlike full spectrum recording which digitally samples the whole event, zero crossing analysis measures the frequency of the sound by counting the oscillations of the waveform around a reference point. In so doing, small files are produced, but the method loses information about amplitude of the waveform, and is generally unable to record harmonics (Corben, n.d.). As such, zero crossing analysis misses factors which can be useful in species identification, particularly where the calls are from acoustically similar species. The resulting sonograms were analyzed by visual inspection (prior to the full transition to fully automated acoustic identification), to identify bat calls to genus level.

#### 2.2.2 Video monitoring

In combination with the automated acoustic recording, video monitoring of entry and exit of bats at “Middle Earth” cave was undertaken on two occasions (August 28, 2017, and 23 September 23, 2017), using a Canon XA10 video recorder working in infrared mode with an infrared light source. The camera was placed just outside the Middle Earth cave entrance, recording into the cave entrance. Recordings were made from 30 min before sunset to 3.5 h after sunset. Numbers of bats entering and leaving the cave were recorded.

#### 2.2.3 Examination of bats in the hand

All bats caught for this study were trapped using a combination of mist nest and harp traps, under a license issued by Natural Resources Wales (license no. 73106c:OTH:SRAB:2017). Catches took place in four different areas of the woodland around the focal cave (“Middle Earth”), during the swarming seasons of 2017 (three nights) and 2018 (three nights, plus one additional night outside the main swarming period). All catches were made within ~100 m of the cave entrance. Bats were only trapped at the cave entrance on two occasions, so as to minimize the possibility of bats in the traps echo locating and being detected by the bat detector at the cave entrance, thereby falsely elevating the number of calls recorded. Alternatively, the presence of the trap may have acted to keep bats away from the cave and therefore reduced the number of calls recorded. Trapping sessions ran from sunset until bat activity declined substantially (in the early hours of the morning). The exceptions were the nights of September 23, 2017, and September 12, 2018, when on both nights trapping terminated at 22.00 GMT even though activity at the time was high.

### 2.3 Meterological data

Hourly temperature and rainfall data for 2017 and 2018 were obtained for the whole study period, from the daily Meteorological Office summaries available at www.metoffice.gov.uk/public/weather/observation/gcnjg1jby.

For the analysis, rainfall was quantified as the percentage of the night (to the nearest 10%) in which rain was detected. In 2017, ambient temperature at midnight (GMT) was the chosen nightly temperature used. For 2018, dusk temperature was also obtained from the Titley Anabat Swift bat detector, and as this proved to be a better predictor of bat activity than midnight temperature (see Section 3), dusk temperature was used in the analysis of 2018 data.

### 2.4 Analysis of data

Analyses were carried out using the statistical software “R” (version 3.2.3, R Core Team, 2016), with methods following Thomas et al. (2017). The MASS package (Venables & Ripley, 2002) and the mgcv package (Wood, 2011) were used to implement for each species a negative binomial generalized additive model (GAM) analysis of the number of echo location calls. Model selection was tailored to identify the detail of seasonal patterns (week‐to‐week variation), by choosing *K*‐values (degree of non‐linearity) that was higher than needed to minimize AIC. These models examined nonlinear temporal (seasonal and/or overnight) variation in calling activity or temperature (Figure 2b). Outputs of all GAM models are presented in the Appendix 1. The results display actual observed activity, rather than activity corrected for the effect of temperature, in order to allow direct comparison with the capture data. Capture data were analyzed using a Chi‐squared approach to test for sex‐ratio differences for each *Myotis* species within and between years, and using negative binomial GAM models to examine seasonal and between‐year variation in capture rate. Results of video monitoring of bat activity at the cave entrance were presented graphically. Data files and R script files for running the analyses are archived in the Data S1.

## 3 RESULTS

### 3.1 The use of the study area by swarming *Myotis* bats

**FIGURE 1:**
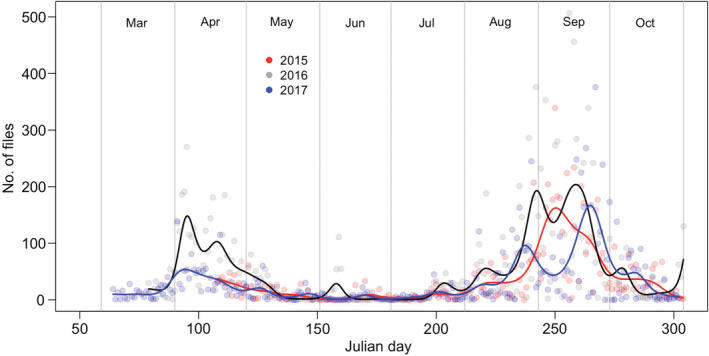
Seasonal variation in the activity of *Myotis* bats (all species combined) at “Hobbit Hole” cave in 2015–2017, measured as the number of zero crossing files, and identified to genus level by visual inspection of the resulting sonograms. Fitted lines show GAM analysis of activity in each year (Table A1). Standard errors not included for the sake of clarity.

The study examined the role of the “Middle Earth” and “Hobbit Hole” caves as swarming sites for *Myotis* bats, as has recently been demonstrated for lesser horseshoe bats *Rhinolophus hipposideros* (Davison & Thomas, 2017). Analysis of zero crossing echolocation files recorded at “Hobbit Hole” cave between 2015 and 2017, and analyzed by visual inspection of the resulting sonograms, shows considerable peaks in *Myotis* bat activity in Spring and during the Autumn swarming season (Figure 1). The peak in the Spring is assumed to be when bats are emerging from hibernation in the caves. The timing of Autumn swarming at this cave (all *Myotis* species combined) varies slightly between the years, but in general lasts from late July to early October. In 2016 and 2017, *Myotis* activity during the Autumn swarming season exhibited a clear double‐peak of activity, with a gap of 1–2 weeks between these peaks. In 2015, the swarming season started later and finished earlier than in the other 2 years, with less overall activity and a less evident double peak, with the primary peak in 2015 falling intermediate in date between the clear double peaks of the other 2 years (Figure 1).

### 3.2 The effectiveness of the Bat Classify software

Different *Myotis* bat species have similar calls, which are often difficult to separate from—and may overlap with—the calls of other *Myotis* species (Barataud, 2015). A confidence level of 80% was selected for the automated identification of species using the Bat Classify software. Below the 80% confidence level, the Bat Classify software frequently gave more than one possible identification for the calls, whereas above the 80% level, too few call sequences were identified to allow a substantial sample size for statistical analysis. Even at this confidence level, many *Myotis* call sequences were not ascribed to an individual species. Indeed, only 27% of calls that were manually identified as *Myotis* type calls were assigned by the automated software to a particular *Myotis* species at the 80% confidence level or above. By contrast, for a more readily identifiable species, 96% of manually identifiable lesser horseshoe bat *Rhinolophus hipposideros* calls were assigned by the software to this species at the 80% confidence level or above.

### 3.3 Results of automated acoustic monitoring

#### 3.3.1 Seasonal variation in activity

**FIGURE 2:**
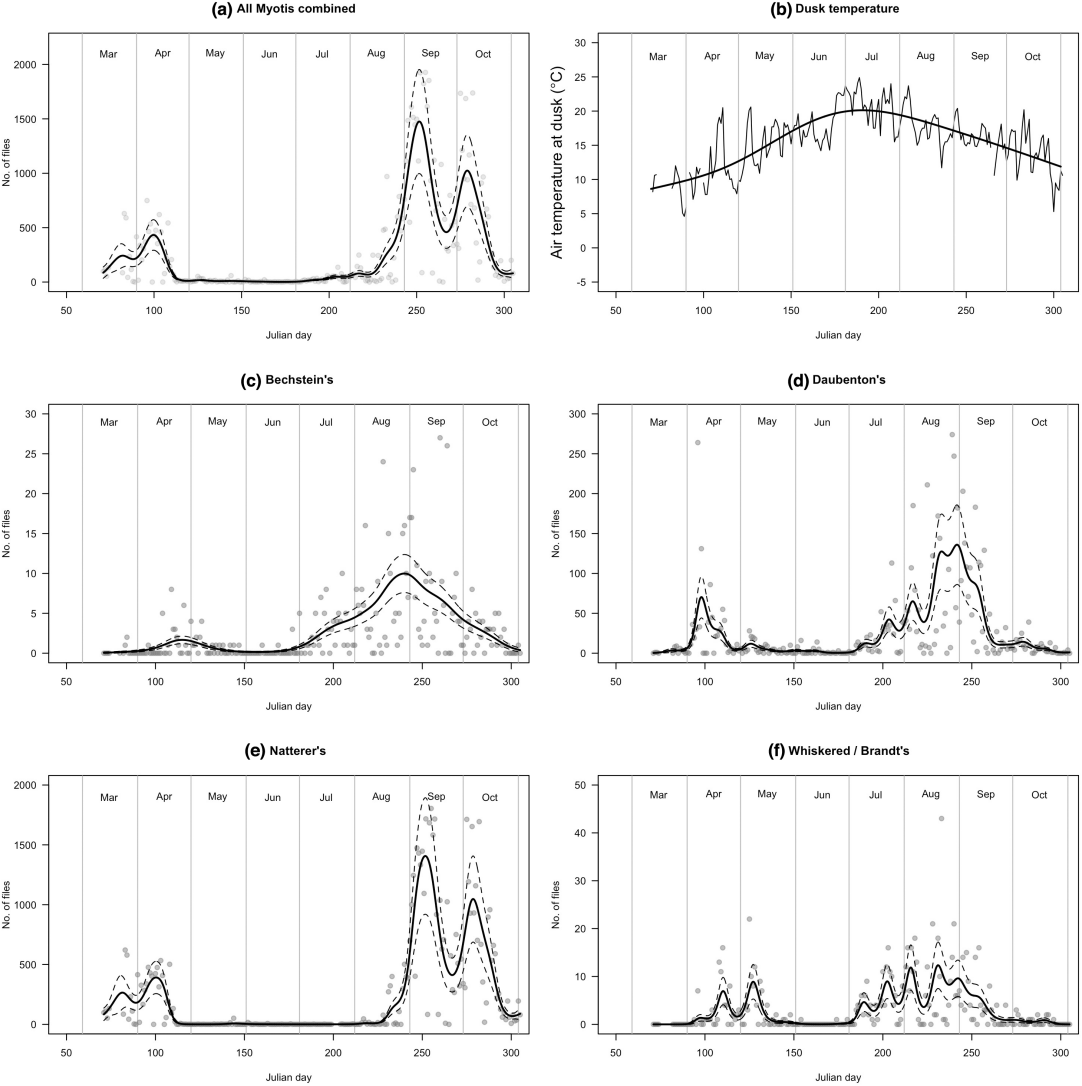
Seasonal variation in the activity of *Myotis* bats at “Middle Earth” cave in 2018. Panel (a) shows all *Myotis* species combined, detected as full spectrum sonograms and identified by automated identification to genus level using the Bat Classify software. The smoothed seasonal pattern (fitted using a GAM, effective degrees of freedom = 5) is shown in addition to the raw data. The temperature data shown in panel (b) were obtained from the mouth of the cave at dusk, and the smoothed seasonal pattern (fitted using a GAM, effective degrees of freedom = 5) is shown in addition to the raw data. Seasonal activity patterns of individual taxa are shown in panels (c)–(f); the smoothed seasonal pattern for each taxon (fitted using a GAM, effective degrees of freedom = 5) is shown in addition to the raw data. For all graphs, the upper and lower smoothed dotted lines show ±1 SE, respectively. The GAM analyses for these seasonal variations are shown in Tables A3 and A4.

Variation in acoustic activity during 2018 clearly shows species differences in seasonal patterns (Figure 2, panels c–f). The acoustic monitoring reveals that Daubenton's bat swarms earlier than Natterer's bat. Bechstein's bat had a much lower number of calls recorded (as would be expected from trapping records, and its lower acoustic amplitude; Barataud, 2015), but is active across the *Myotis* swarming period. In the case of Brandt's/whiskered bats, which cannot be reliably separated by acoustic analysis, there are two main periods of activity: early and late in the swarming season. The catch data (Figure 4) show that Brandt's bat appears earlier in the season than whiskered bat, suggesting that the earlier acoustic peak in Figure 2, panel f, is primarily composed of Brandt's bats and the later peak is primarily composed of whiskered bats. Figure 2, panel e shows bimodal peaks of calling activity in 2018 for Natterer's bat. The pattern for other species is less clear, possibly due to the lower counts involved.

#### 3.3.2 Activity throughout the night

**FIGURE 3:**
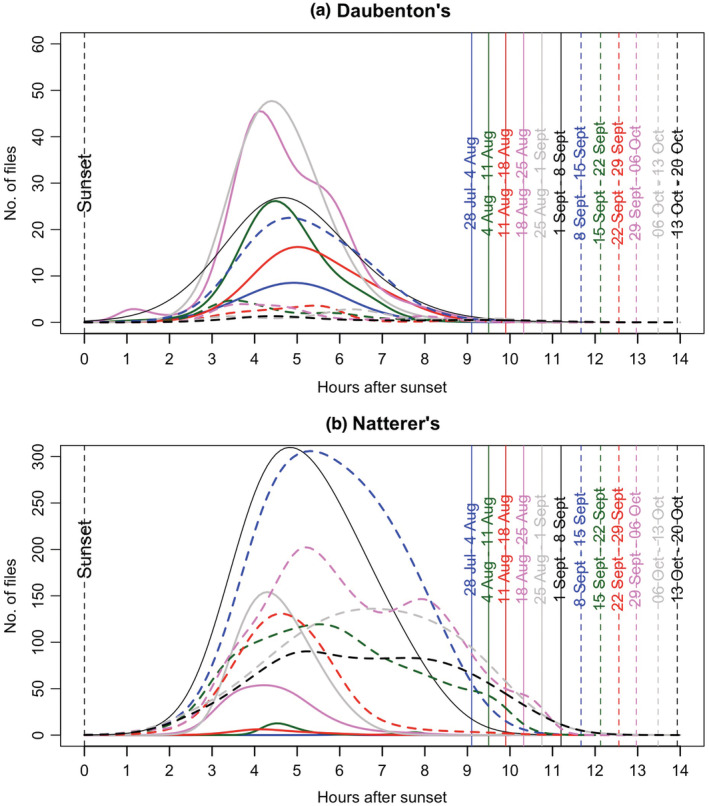
Hourly activity of different *Myotis* species across the night, across the 2018 peak swarming period. The vertical lines indicate the changing night lengths throughout the study period, and the colors indicate respective weeks. (a) Overnight distribution of *Myotis nattereri* calls. (b) Overnight distribution of *Myotis daubentonii* calls. Vertical lines and colors represent mean time of sunrise during each week of the study period. Fitted lines show GAM analysis for each week throughout the swarming period (Tables A5 and A6). Standard errors are not shown for the sake of clarity.

The activity of different *Myotis* bat species, as measured by acoustic monitoring, varied both through the night, and through the season. Figure 3 shows nightly activity of Natterer's and Daubenton's bats from dusk, for each week of significant activity. Peak overnight activity for these two species varies slightly between weeks but occurs typically about 4 h after dusk. Within the swarming period for each individual species, there was a tendency for activity to continue further into the night as nights became longer, later in the year. There were too few hourly data points for Brandt's/whiskered and Bechstein's bats to analyze overnight patterns of activity in this way.

### 3.4 Capture data

Trapping of bats in woodland adjacent to “Middle Earth” cave could only be undertaken on a 3–4 nights in each swarming season (to minimize disturbance). To obtain sufficient sample size for each taxon, catches were pooled for analysis across 2013–2018. Overall, the calendar order of peak swarming activity for each species among the manually captured bats (aggregated from 2013–2018) follows the same order as individually identifiable species detected acoustically (i.e., Daubenton's > Bechsteins > Natterer's).

The captured sample of *Myotis* bats showed a strongly male‐biased sex ratio. For all species with a sample size large enough to make a species‐specific sex ratio comparison (Bechstein's, Natterer's, Whiskered and Daubenton's), this male bias was statistically significant, and this male‐bias did not vary significantly between years (Table 1).

**TABLE 1:** Sex ratios among *Myotis* bats captured at “Middle Earth” cave and in adjacent woodland in 2017 and 2018, using mist nets and harp traps.

Species	2017			2018			Total 2017–18			Between year sex ratio comparison
	M:F	% Male	*p*	M:F	% Male	*p*	M:F	% Male	*p*	*p*
*M. bechsteinii*	19:2	90.5	.006	21:5	80.8	.040	40:7	85.1	.0004	.400
*M. nattereri*	39:9	81.2	.002	62:5	92.5	<.0001	101:14	87.8	<.0001	.090
*M. mystacinus*	35:11	76.1	.020	17:4	81.0	.050	52:15	77.6	.001	.800
*M. brandtii*	4:3	74.3	–	1:0	100.0	–	5:3	62.5	–	–
*M. daubentonii*	33:5	86.8	.001	13:1	92.9	.030	46:6	88.5	<.0001	1.000

*Note*: Male: female ratios were compared using Fisher's exact test for sex‐ratio bias (null hypothesis of 1:1), and for differences in sex ratio between 2017 and 2018.

**FIGURE 4:**
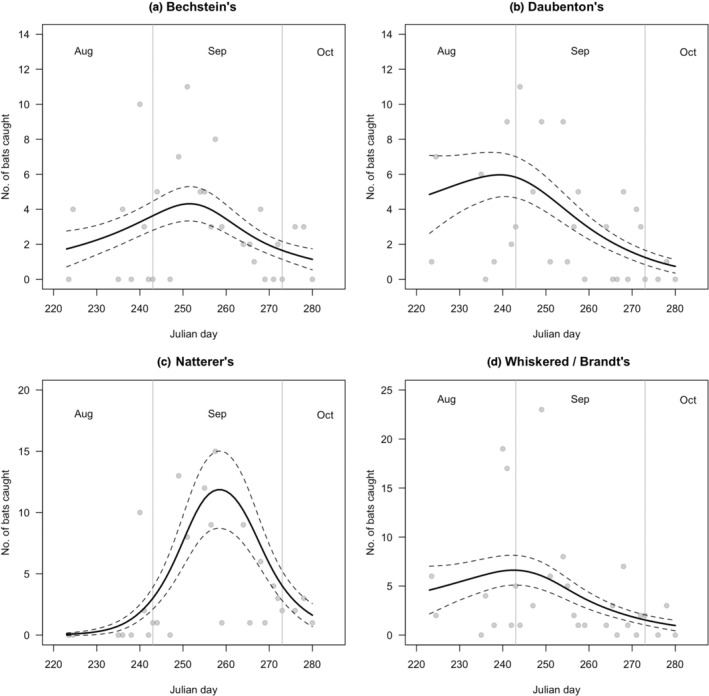
Catches of different *Myotis* species in 2017–2018. Captures are plotted against calendar date, but in each year, the timing of peak activity was different. Solid lines represent model fitted lines from the GAM model for each taxon described in Table A2 (negative binomial error family, log‐link function, maximum *k*‐value = 40). Dashed lines represent ±1SE.

Catches in 2017 and 2018 showed that the peak of swarming activity of Brandt's bat occurs earlier in the season than the peak swarming activity of whiskered bats (Figure 4) but at the cave itself, very little acoustic activity was detected that could be attributed to these two species which are hard to differentiate acoustically. It is unlikely that this was due to the limitations of the Bat Classify software, as in the adjacent woodland 12.6% of the Anabat Walkabout's 87 recorded sound files were identified by the Bat Classify software as either whiskered or Brandt's bats. This shows that the calls of these two species were detectable and successfully identified as one or other of this species‐pair by the software in the woodland; hence, there was probably a genuine lack of activity by these two species at the cave.

### 3.5 Video monitoring of use of the cave by bats

**FIGURE 5:**
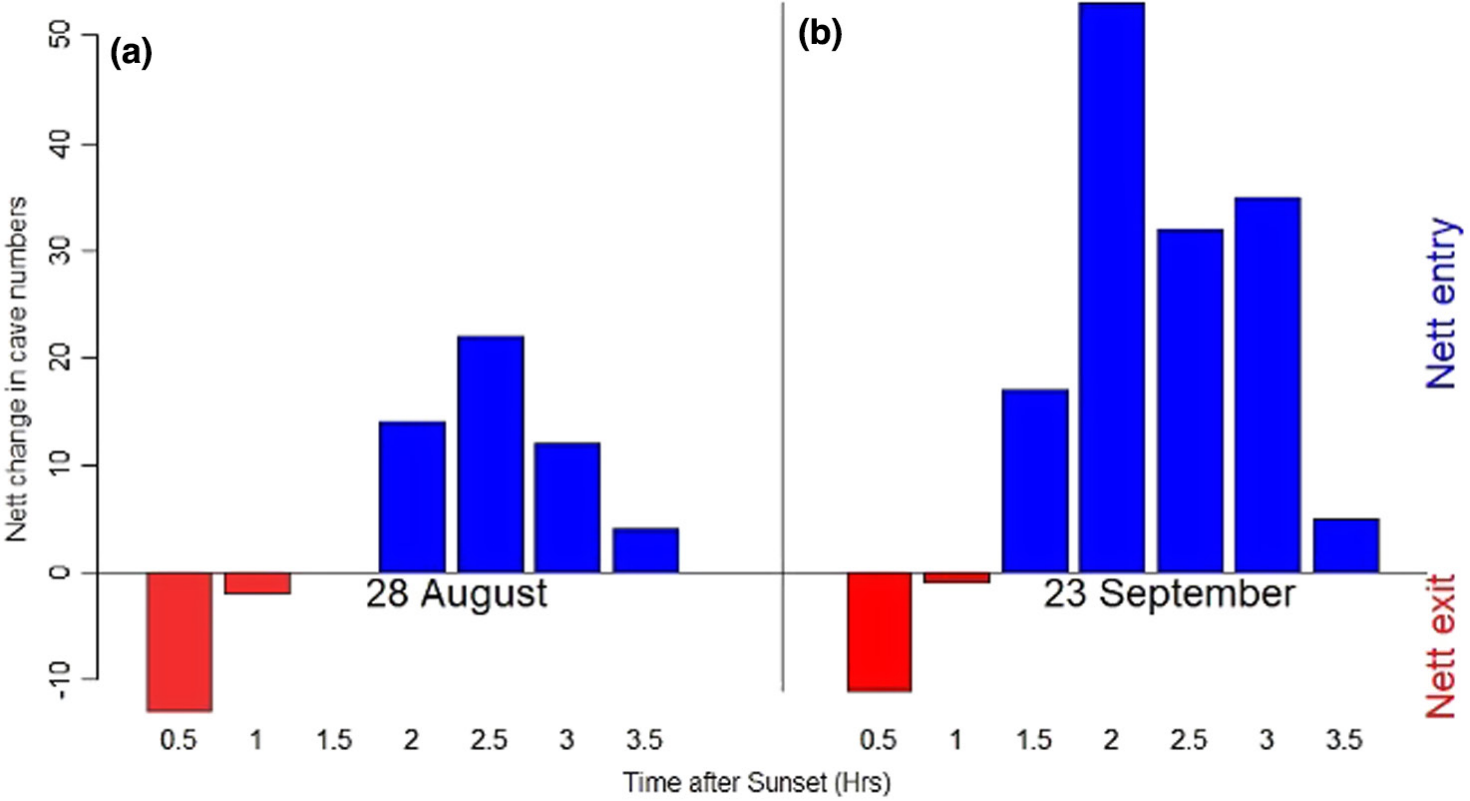
Video‐evidence of bats entering and leaving the focal cave on August 28, 2017, and September 28, 2017, from half an hour before sunset, until 3.5 h after sunset.

It was not possible to identify the bats to species‐level using the video recordings; some were shown from analysis of simultaneously recorded heterodyne sound files to have been *Rhinolophus hipposideros* (and therefore removed from the analysis), whereas others were *Myotis* species. Overall, bat activity (number of entries and exits) detected by infrared video monitoring on September 23, 2017, was greater than that recorded earlier on August 28, 2017 (Figure 5). On both dates, bats initially left the cave (where many had been roosting) following sunset. Bats later returned to the cave, many exhibiting “chase” sequences (one bat following another) which were visible on the infrared video recordings. From 1.5 to 2 h after sunset, there was a net influx of bats into the cave, continuing until 3.5 h after sunset.

## 4 DISCUSSION

Developments in bat detection technology (high‐quality ultrasonic recording devices) and acoustic identification software (using acoustic features of bat calls to separate different species) have enabled more detailed recording of bat echolocation calls, and the automated identification of the species emitting the call. Unfortunately, however, independent testing of these systems reveals variable effectiveness, with individual researchers' manual identification of calls from sonograms also showing imperfect species recognition (Clement et al., 2014; Russo & Voigt, 2016; Rydell et al., 2017). This is hardly surprising given the variability in call structure shown by individual species of bats in different habitats, and the inherent similarity between the calls of some species (Barataud, 2015). In this study, however, despite the imperfections of acoustic monitoring with automated identification, and manual trapping, the two methods revealed remarkably similar activity patterns. Furthermore, acoustic information is available constantly throughout every night of the swarming season, whereas trapping can only be undertaken infrequently during the swarming season, to minimize the potential disturbance caused to the bats by capture and handling. Both methods have their advantages and disadvantages. In the case of trapping, radio‐tracking data for male bats suggest that they can learn to avoid traps within the swarming season (Kunz & Brock, 1975). This “trap‐shyness” would affect the numbers captured on subsequent nights and impair population estimation from data on captures and recaptures within the same year. Additionally, some species, demographic groups, or individuals may be better at avoiding traps in the first place, leading to potential bias in population estimates.

While acoustic monitoring is most unlikely to affect a bat's activity, different species have different intensities of call (Meyer et al., 2011; Neuweiler, 1989; Richardson et al., 2019). This leads to a microphone detecting some species at greater distances than others, giving rise to biases when attempting to compare abundance between species by this method. Additionally, different species are likely to have different probabilities of identification from analysis of their echolocation calls (Barataud, 2015; Clement et al., 2014; Murray et al., 2007; Rydell et al., 2017; Tuneu‐Corrala et al., 2020). Consequently, absolute activity levels of one species determined by this method cannot therefore be compared directly with those of another species. Absolute abundance is therefore unlikely to be measured effectively by either method, but both have their different advantages in determining changes in relative abundance within taxa, for example across the swarming season and across the night.

Capture gives the opportunity to identify bats in the hand, to note their sex, identify sex ratio biases (Table 1) and in the case of males, to record their reproductive status. In the case of females, it is usually possible to observe whether they have given previously birth, and sometimes whether the individual is a juvenile. By contrast, acoustic monitoring is of no use in determining sex or breeding status of the population. It is, however, useful in determining seasonal (nightly, Figure 2) and overnight (hourly, Figure 3) activity patterns, which have been shown in the present study to vary substantially throughout the swarming period. Video monitoring of bats entering and leaving the cave, combined with acoustic detection, provides additional information by revealing that *Myotis* bats increasingly accumulate within the cave during the first part of the night (net inwards flux of bats into the cave), following an initial exodus (net outwards flux) at dusk (Figure 5).

Individual species within the *Myotis* genus may have different rates of correct species assignment, as some species may be easier for the software to recognize from their acoustic signature than others, and some species may more frequently give atypical calls at the swarming site as opposed to the calls that they make in their more usual habitat. Bats typically have the ability to vary their call structure to match the challenges of different environments (Barataud, 2015; Russ, 2021), and thus, it is possible that some of the calls recorded during swarming are atypical. Indeed, we already know that bat social calls recorded during swarming can be different to those observed at other times of the year (Middleton, 2022). Manually checking several thousand files of echolocation data each night through multiple seasons would prove to be a challenge; hence, automatic classification of the calls is the only practical way forward, even though neither approach is likely to be 100% accurate (Russo & Voigt, 2016; Rydell et al., 2017). Nevertheless, the aggregated automatically identified acoustic records correspond well with the capture data (Figures 2 and 4).

The notable bimodal seasonal peak in swarming activity of *Myotis nattereri*, shown in the present study (Figure 2), is unlikely to have been detected by sporadic trapping (cf. Figure 4). A possible explanation for this bimodality is that swarming has multiple functions; for example, mating could account for the first peak, and prehibernation activity could account for the second peak—but this interpretation clearly needs further investigations. There is a suggestion of similar bimodal activity patterns for the other *Myotis* species, although the lower numbers recorded prevented a firm conclusion in these cases.

This study has demonstrated that the use of acoustic monitoring adds significantly to data obtained by catching bats at swarming periods, and for some research questions may provide sufficient or additional information without disturbing the animals. The automated acoustic identification software has its limitations in consistently identifying individual species' echolocation calls, but, over the swarming season, patterns of activity shown by acoustic monitoring are remarkably consistent with those derived from trapping. In contrast to capture‐based methods, automated acoustic monitoring allows a far more detailed analysis of temporal (seasonal and overnight) variation. If the efficiency of the algorithms used to identify bats can be improved, this technique for quantifying seasonal variation in bat activity, including swarming activity, will become even more effective. Overall, neither trapping nor acoustic identification alone provide a fully comprehensive and accurate method of studying bat swarming behavior, but the use of both methods, in parallel with additional approaches such as infrared video monitoring of cave entrances, represents a powerful combined approach, providing a deeper understanding of bat behavior than can be gained using either method individually.

It is postulated that swarming behavior is used by bats for mating and/or as a way of identifying or checking on hibernation sites. As such, these swarming sites are pf particular importance to the lives of bats and their conservation (Parsons, Jones, & Greenaway, 2003; Parsons, Jones, Davidson‐Watts, et al., 2003; Rivers et al., 2006; van Shaik et al., 2015). The present study provides an automated method of monitoring bat activity at swarming sites, which is fundamental to enabling practitioners to ensure protection of these sites and the species that use them.

## AUTHOR CONTRIBUTIONS

**Robert J. Thomas:** Conceptualization (equal); formal analysis (equal); investigation (equal); methodology (equal); resources (equal); software (equal); validation (equal); visualization (equal); writing – original draft (equal); writing – review and editing (equal). **Stephen P. Davison:** Conceptualization (equal); data curation (equal); formal analysis (equal); investigation (equal); methodology (equal); resources (equal); software (equal); validation (equal); visualization (equal); writing – original draft (equal); writing – review and editing (equal).

### OPEN RESEARCH BADGES

This article has earned Open Data and Open Materials badges. Data and materials are available at https://datadryad.org/stash/dataset/doi:10.5061/dryad.66t1g1k35?.

## Supporting information


Data S1

## Data Availability

Data and analysis script files are archived in the Dryad data repository: Thomas and Davison (2022). https://datadryad.org/stash/dataset/doi:10.5061/dryad.66t1g1k35?

## APPENDIX 1

**TABLE A1:** A generalized additive model to explain variation in nightly abundance of different taxa of *Myotis* bats detected at “Hobbit Hole” cave in 2015–17 using automated acoustic monitoring.

Independent variables	Category	df/edf	Parameter estimate	SE	Test statistic	*p*
**Parametric terms**					***Z***	
Intercept		1	2.952	0.689	4.284	<.0001
Year (reference category = 2015)	2016	1	0.030	0.698	0.043	.966
	2017	1	−0.156	0.693	−0.225	.822
**Smoothed terms**					**Chi** ^**2**^	
	Julian day × 2015	18.94			278.4	<.0001
	Julian day × 2016	28.23			429.5	<.0001
	Julian day × 2017	27.29			292.2	<.0001

*Note*: The model used a negative binomial error family (scale = 1) and a log‐link function. Max *k* = 40. Overdispersion statistic = 1.061, deviance explained = 61.7%. The prediction plot from this model is shown in Figure 1.

Abbreviation: edf, effective degrees of freedom.

**TABLE A2:** Generalized additive models to explain variation in nightly abundance of different taxa of *Myotis* bats captured at “Middle Earth” cave in 2018.

Taxon	Independent variables	df/edf	Parameter estimate	SE	Test statistic	*p*
(i) Bechstein's bat	**Parametric term**				***Z***	
	Intercept	1	0.982	0.159	6.175	<.0001
	**Smoothed term**				**Chi** ^**2**^	
	Julian day	2.421			6.529	.0892
(ii) Daubenton's bat	**Parametric term**				***Z***	
	Intercept	1	1.116	0.160	6.991	<.0001
	**Smoothed term**				**Chi** ^**2**^	
	Julian day	2.120			14.870	.00133
(iii) Natterer's bat	**Parametric term**				***Z***	
	Intercept	1	1.998	0.203	5.914	<.0001
	**Smoothed term**				**Chi** ^**2**^	
	Julian day	2.795			25.040	<.0001
(iv) Brandt's /Whiskered bat	**Parametric term**				***Z***	
	Intercept	1	1.266	0.170	7.438	<.0001
	**Smoothed term**				**Chi** ^**2**^	
	Julian day	2.199			0.006	

*Note*: Each model used a negative binomial error family (scale = 1) and a log‐link function. Max *k* = 40.

*Bechstein's bat*. Overdispersion statistic = 1.907, deviance explained = 15.9%. The prediction plot from this model is shown in Figure 4a.
*Daubenton's bat*. Overdispersion statistic = 1.922, deviance explained = 26.9%. The prediction plot from this model is shown in Figure 4b.
*Natterer's bat*. Overdispersion statistic = 1.673, deviance explained = 45.9%. The prediction plot from this model is shown in Figure 4c.
*Brandt's/Whiskered bat*. Overdispersion statistic = 1.642, deviance explained = 25.5%. The prediction plot from this model is shown in Figure 4d.

Abbreviation: edf, effective degrees of freedom.

**TABLE A3:** A generalized additive model to explain variation in nightly activity of different taxa of *Myotis* bats (all taxa combined) at Middle Earth cave in 2018.

Independent variables	Category	df/edf	Parameter estimate	SE	Test statistic	*p*
**Parametric term**					***Z***	
Intercept		1	4.205	0.0688	61.15	<.0001
**Smoothed term**					**Chi** ^**2**^	
	Julian day	23.94			785.6	<.0001

*Note*: The model used a negative binomial error family (scale = 1) and a log‐link function. Max *k* = 40. Overdispersion statistic = 1.343, deviance explained = 69.6%. The prediction plot from this model is shown in Figure 4, panel (a).

Abbreviation: edf, effective degrees of freedom.

**TABLE A4:** A generalized additive model to explain variation in nightly abundance of different taxa of *Myotis* bats detected at Middle Earth cave in 2018, using automated acoustic monitoring.

Independent variables	Category	df/edf	Parameter estimate	SE	Test statistic	*p*
**Parametric terms**					***Z***	
Intercept		1	0.322	0.107	3.019	.003
Species (reference category = Bechstein's)	Daubenton's	1	1.985	0.131	15.199	<.0001
	Natterer's	1	2.640	0.138	19.113	<.0001
	Whiskered/Brandt's	1	−0.631	0.402	−1.567	.117
**Smoothed terms**					**Chi** ^**2**^	
	Julian day × Bechstein's	10.17			160.7	<.0001
	Julian day × Daubenton's	29.95			387.0	<.0001
	Julian day × Natterer's	25.51			1153.4	<.0001
	Julian day × Whiskered /Brandt's	26.95			151.9	<.0001

*Note*: The model used a negative binomial error family (scale = 1) and a log‐link function. Max *k* = 40. Overdispersion statistic = 1.208, deviance explained = 82%. The prediction plot from this model is shown in Figure 4 (panels c–f).

Abbreviation: edf, effective degrees of freedom.

**TABLE A5:** A generalized additive model to explain variation in hourly abundance of *Daubenton's bats*, detected at Middle Earth cave in 2018, using automated acoustic monitoring.

Independent variables	Category	df/edf	Parameter estimate	SE	Test statistic	*p*
**Parametric terms**		**df**			***Z***	
Intercept		1	−0.739	0.573	−1.290	.197
Week (reference category = Week 5)	Week 1	1	−0.713	0.816	−0.873	.383
	Week 2	1	+1.565	1.035	1.512	.131
	Week 3	1	−0.089	0.770	−0.116	.908
	Week 4	1	+0.777	1.434	0.542	.588
	Week 6	1	+1.499	0.613	2.446	**.015**
	Week 7	1	+0.806	0.638	1.264	.206
	Week 8	1	−0.897	0.835	−1.074	.283
	Week 9	1	−2.054	1.538	−1.336	.182
	Week 10	1	−1.729	1.176	−1.470	.142
	Week 11	1	−0.993	0.948	−1.047	.295
	Week 12	1	−0.838	0.688	−1.219	.223
	Week 13	1	−4.878	2.998	−1.627	.104
	Week 14	1	−9.841	22.635	−0.435	.6637
**Smoothed terms**		**edf**			**Chi** ^**2**^	
	Hour × Week 1	3.178			56.091	<.0001
	Hour × Week 2	7.232			100.614	<.0001
	Hour × Week 3	3.980			68.257	<.0001
	Hour × Week 4	8.995			104.527	<.0001
	Hour × Week 5	4.330			118.014	<.0001
	Hour × Week 6	3.518			109.619	
	Hour × Week 7	4.472			105.544	
	Hour × Week 8	6.120			34.072	<.0001
	Hour × Week 9	6.782			31.353	.0001
	Hour × Week 10	6.428			26.687	.0005
	Hour × Week 11	7.620			21.382	.0127
	Hour × Week 12	5.053			16.567	.0178
	Hour × Week 13	3.204			10.979	.0329
	Hour × Week 14	4.809			2.988	.831

*Note*: The model used a negative binomial error family and a log‐link function. The prediction plot from this model is shown in Figure 5a. Max *k* = 15. Overdispersion statistic = 0.974, deviance explained = 71.6%.

Abbreviation: edf, effective degrees of freedom.

**TABLE A6:** A generalized additive model to explain variation in hourly abundance of *Natterer's bats*, detected at Middle Earth cave in 2018, using automated acoustic monitoring.

Independent variables	Category	df/edf	Parameter estimate	SE	Test statistic	*p*
**Parametric terms**		**df**			***Z***	
Intercept		1	2.5575	0.221	11.576	<.0001
Week (reference category = Week 7)	Week 1	1	−20.058	2.230	−0.393	.694
	Week 2	1	−12.048	27.384	−3.325	.660
	Week 3	1	−6.223	1.872	−3.325	.0009
	Week 4	1	−3.967	1.193	−3.326	.0009
	Week 5	1	−6.387	2.230	−2.865	.00417
	Week 6	1	−0.473	0.340	−1.393	.164
	Week 8	1	−0.658	0.391	−1.685	.092
	Week 9	1	−1.439	0.349	−4.121	<.0001
	Week 10	1	−0.412	0.522	−0.789	.430
	Week 11	1	0.438	0.253	1.736	.082
	Week 12	1	0.153	0.256	0.597	.550
	Week 13	1	−1.574	0.273	−5.777	<.0001
	Week 14	1	−1.744	0.277	−6.302	<.0001
**Smoothed terms**		**edf**			**Chi** ^**2**^	
	Hour × Week 1	4.240			2.179	.754
	Hour × Week 2	7.675			24.682	.0018
	Hour × Week 3	4.042			33.654	<.0001
	Hour × Week 4	5.640			111.238	<.0001
	Hour × Week 5	4.600			126.809	<.0001
	Hour × Week 6	4.874			253.107	<.0001
	Hour × Week 7	6.309			265.450	<.0001
	Hour × Week 8	7.810			162.098	<.0001
	Hour × Week 9	6.160			198.349	<.0001
	Hour × Week 10	10.781			140.149	<.0001
	Hour × Week 11	6.049			212.760	<.0001
	Hour × Week 12	6.561			178.182	<.0001
	Hour × Week 13	5.311			127.143	<.0001
	Hour × Week 14	7.562			79.144	<.0001

*Note*: The model used a negative binomial error family and a log‐link function. The prediction plot from this model is shown in Figure 5b. Max *k* = 15. Overdispersion statistic = 1.248, deviance explained = 77.2%.

Abbreviation: edf, effective degrees of freedom.
