# Supplementary material for: Seasonal swarming behavior of Myotis bats revealed by integrated monitoring, involving passive acoustic monitoring with automated analysis, trapping, and video monitoring
Source: Ecol Evol. 2022 Sep 23;12(9):e9344. doi: 10.1002/ece3.9344 (PMC9502064; doi:10.1002/ece3.9344)
Supplement: Supplementary file 1 — Data S1 [file ECE3-12-e9344-s001.docx]

#### Thomas & Davison -archived script ####

#.####

# Set working directory ####

setwd("~/Dropbox/Stephen Davison bats/Paper 2")

#.####

# Figure 1 (Myotis activity, Hobbit Hole Cave 2015-17) ####

dframe1 <- read.csv("Myotis Hobbit Hole 2015-17.csv", stringsAsFactors = T)

summary(dframe1)

names(dframe1)

dframe1$Fyear <- as.factor(dframe1$year) # Turn year into a factor

#...Model of Myotis activity 2015-17 ####

library(mgcv)

library(MASS)

model1 <- gam(no ~ s(J.day, by = Fyear, fx = F, k=40) + Fyear,

data=dframe1, family=negbin(link="log", theta=1<100))

AIC(model1) # 5364.503

summary(model1)

names(model1)

# gam.check(model1) # model validation graphs

# Calculate the overdispersion statistic.

# Overdispersion = residual devaince / residual d.f.

overdispersion <- model1$deviance / model1$df.residual

overdispersion # 1.06101

#...Plot seasonal variation for each year ####

?png

jpeg(file = "Figure 1", quality = 100, res = 250, # activeate these lines to create the hi-res multi-panel plot

width = 2500, height = 1500)

par(mfrow = c(1,1))

plot(no ~ J.day, col="white", data = dframe1,

xlim = c(50,304),

ylab = "No. of files", xlab = "Julian day", las = 1)

# Add monthly lines

abline(v = 31, col="grey") # End of Jan

abline(v= 31+28, col="grey") # end of Feb

text(31+28+15, 490, "Mar")

abline(v= 31+28+31, col="grey") # end of Mar

text(31+28+31+15, 490, "Apr")

abline(v= 31+28+31+30, col="grey") # end of April

text(31+28+31+30+15, 490, "May")

abline(v= 31+28+31+30+31, col="grey") # end of May

text(31+28+31+30+31+15, 490, "Jun")

abline(v= 31+28+31+30+31+30, col="grey") # end of June

text(31+28+31+30+31+30+15, 490, "Jul")

abline(v= 31+28+31+30+31+30+31, col="grey") # end of July

text(31+28+31+30+31+30+31+15, 490, "Aug")

abline(v= 31+28+31+30+31+30+31+31, col="grey") # end of Aug

text(31+28+31+30+31+30+31+31+15, 490, "Sep")

abline(v= 31+28+31+30+31+30+31+31+30, col="grey") # end of Sept

text(31+28+31+30+31+30+31+31+30+15, 490, "Oct")

abline(v= 31+28+31+30+31+30+31+31+30+31, col="grey") # end of Oct

points(no ~ J.day, data = dframe1, subset= dframe1$year==2015,

pch=19, col=rgb(1,0,0,0.2))

points(no ~ J.day, data = dframe1, subset= dframe1$year==2016,

pch=19, col=rgb(0.5,0.5,0.5,0.2))

points(no ~ J.day, data = dframe1, subset= dframe1$year==2017,

pch=19, col=rgb(0,0,1,0.2))

legend(125,450, box.col = "white", pch = 19,

legend = c("2015", "2016", "2017"),

col=c("red", "dark grey", "blue"))

# Plot 2015

summary(dframe1$J.day[dframe1$Fyear=="2015"])

pdat<-expand.grid(J.day=seq(108,304,0.1), Fyear="2015")

pdat

pred<-predict(model1,newdata=pdat,na.rm=T,type="response",se.fit=T)

pred

predframe<-data.frame(pdat,preds=pred$fit,se=pred$se.fit)

predframe

lines(predframe$preds ~ predframe$J.day, col="red", lwd =2)

# Plot 2016

summary(dframe1$J.day[dframe1$Fyear=="2016"])

pdat<-expand.grid(J.day=seq(79,304,0.1), Fyear="2016")

pdat

pred<-predict(model1,newdata=pdat,na.rm=T,type="response",se.fit=T)

pred

predframe<-data.frame(pdat,preds=pred$fit,se=pred$se.fit)

predframe

lines(predframe$preds ~ predframe$J.day, col="black", lwd = 2)

# Plot 2017

summary(dframe1$J.day[dframe1$Fyear=="2017"])

pdat<-expand.grid(J.day=seq(64,302,0.1), Fyear="2017")

pdat

pred<-predict(model1,newdata=pdat,na.rm=T,type="response",se.fit=T)

pred

predframe<-data.frame(pdat,preds=pred$fit,se=pred$se.fit)

predframe

lines(predframe$preds ~ predframe$J.day, col="blue", lwd=2)

dev.off()

#.####

# Figure 4 (Myotis activity, Middle Earth Cave 2018) ####

dframe1 <- read.csv("Activity Middle Earth 2018_Final.csv", stringsAsFactors = T)

names(dframe1)

summary(dframe1)

summary(dframe1$Species)

dframe1$J.day <- dframe1$count + 11 # Create Julian day variable

#.####

# Compiling total Myotis vs. Week ####

summary(dframe1)

Daub <- subset(dframe1, Species == "dau")

summary(Daub)

Becs <- subset(dframe1, Species == "Bec")

summary(Becs)

Nats <- subset(dframe1, Species == "Nat")

summary(Nats)

Whisk <- subset(dframe1, Species == "WB")

summary(Whisk)

Myotis <- Daub$No + Becs$No + Nats$No + Whisk$No

J.day <- Daub$J.day

#...Model of Total Myotis activity 2018 ####

library(mgcv); library(MASS)

model1 <- gam(Myotis ~ s(J.day, fx = F, k=40), # k=25 minimises AIC

family=negbin(link="log", theta=1<100))

summary(model1)

AIC(model1)

# Calculate the overdispersion statistic.

# Overdispersion = residual devaince / residual d.f.

overdispersion <- model1$deviance / model1$df.residual

overdispersion # 1.342698

# Set up multi-panel plot ####

jpeg(file = "Figure 4", quality = 100, res = 250,

width = 3000, height = 3000)

par(mfrow = c(3,2))

# (i) total Myotis activity 2018 ####

plot(Myotis ~ J.day, las =1,

xlim = c(50,304),

ylim = c(0,2200),

xlab = "Julian day",

ylab = "No. of files",

pch = 19, col=rgb(0.5,0.5,0.5,0.2),

main="(i) All Myotis combined")

# Add monthly lines

abline(v = 31, col="grey") # End of Jan

abline(v= 31+28, col="grey") # end of Feb

text(31+28+15, 2100, "Mar")

abline(v= 31+28+31, col="grey") # end of Mar

text(31+28+31+15, 2100, "Apr")

abline(v= 31+28+31+30, col="grey") # end of April

text(31+28+31+30+15, 2100, "May")

abline(v= 31+28+31+30+31, col="grey") # end of May

text(31+28+31+30+31+15, 2100, "Jun")

abline(v= 31+28+31+30+31+30, col="grey") # end of June

text(31+28+31+30+31+30+15, 2100, "Jul")

abline(v= 31+28+31+30+31+30+31, col="grey") # end of July

text(31+28+31+30+31+30+31+15, 2100, "Aug")

abline(v= 31+28+31+30+31+30+31+31, col="grey") # end of Aug

text(31+28+31+30+31+30+31+31+15, 2100, "Sep")

abline(v= 31+28+31+30+31+30+31+31+30, col="grey") # end of Sept

text(31+28+31+30+31+30+31+31+30+15, 2100, "Oct")

abline(v= 31+28+31+30+31+30+31+31+30+31, col="grey") # end of Oct

# Add Myotis fitted lines

pdat<-expand.grid(J.day=seq(71,305,0.1))

pdat

pred<-predict(model1,newdata=pdat,na.rm=T,type="response",se.fit=T)

pred

predframe<-data.frame(pdat,preds=pred$fit,se=pred$se.fit)

predframe

lines(predframe$preds ~ predframe$J.day, lwd =2)

lines(predframe$preds + predframe$se ~ predframe$J.day, lty=2)

lines(predframe$preds - predframe$se ~ predframe$J.day, lty=2)

#.####

# (ii) Dusk temperature data ####

plot(Temp ~ J.day, ylab="", xlab="Julian day",

ylim = c(-5,30),

xlim = c(50,304),

las=1,

col="white", data=dframe1,

main="(ii) Dusk temperature")

lines(Temp ~ J.day, data=dframe1)

mtext (expression(paste("Air temperature at dusk (", degree,"C)")),

side=2,line=2.5)

# Add monthly lines

abline(v = 31, col="grey") # End of Jan

abline(v= 31+28, col="grey") # end of Feb

text(31+28+15, 28, "Mar")

abline(v= 31+28+31, col="grey") # end of Mar

text(31+28+31+15, 28, "Apr")

abline(v= 31+28+31+30, col="grey") # end of April

text(31+28+31+30+15, 28, "May")

abline(v= 31+28+31+30+31, col="grey") # end of May

text(31+28+31+30+31+15, 28, "Jun")

abline(v= 31+28+31+30+31+30, col="grey") # end of June

text(31+28+31+30+31+30+15, 28, "Jul")

abline(v= 31+28+31+30+31+30+31, col="grey") # end of July

text(31+28+31+30+31+30+31+15, 28, "Aug")

abline(v= 31+28+31+30+31+30+31+31, col="grey") # end of Aug

text(31+28+31+30+31+30+31+31+15, 28, "Sep")

abline(v= 31+28+31+30+31+30+31+31+30, col="grey") # end of Sept

text(31+28+31+30+31+30+31+31+30+15, 28, "Oct")

abline(v= 31+28+31+30+31+30+31+31+30+31, col="grey") # end of Oct

# K=6 temperature model

modeltemp <- gam(Temp ~ s(J.day,fx=T,k=6),

family=gaussian,

link=identity,

na.action=na.exclude,

data=dframe1)

pdat<-expand.grid(J.day=seq(70,304))

pdat

pred<-predict(modeltemp,newdata=pdat,na.rm=T,type="response",se.fit=T)

pred

predframe<-data.frame(pdat,preds=pred$fit,se=pred$se.fit)

predframe

#adding predicted value lines to existing graph

lines(predframe$preds~predframe$J.day,lwd=2,col="black")

#.####

#...Multi-species model of Myotis activity 2018 ####

library(mgcv)

library(MASS)

model1 <- gam(No ~ s(J.day, by = Species, fx = F, k=40) + Species,

data=dframe1, family=negbin(link="log", theta=1<100))

AIC(model1) # 5232.252

summary.gam(model1)

model1

# Calculate the overdispersion statistic.

# Overdispersion = residual devaince / residual d.f.

overdispersion <- model1$deviance / model1$df.residual

overdispersion # 1.207885

#...(iii) Bechstein's ####

plot(No ~ J.day, col="white",

data = dframe1,

ylim=c(0,30),

xlim=c(50,300),

ylab = "No. of files",

xlab = "Julian day",

las = 1,

main="(iii) Bechstein's")

# Add monthly lines

abline(v = 31, col="grey") # End of Jan

abline(v= 31+28, col="grey") # end of Feb

text(31+28+15, 29, "Mar")

abline(v= 31+28+31, col="grey") # end of Mar

text(31+28+31+15, 29, "Apr")

abline(v= 31+28+31+30, col="grey") # end of April

text(31+28+31+30+15, 29, "May")

abline(v= 31+28+31+30+31, col="grey") # end of May

text(31+28+31+30+31+15, 29, "Jun")

abline(v= 31+28+31+30+31+30, col="grey") # end of June

text(31+28+31+30+31+30+15, 29, "Jul")

abline(v= 31+28+31+30+31+30+31, col="grey") # end of July

text(31+28+31+30+31+30+31+15, 29, "Aug")

abline(v= 31+28+31+30+31+30+31+31, col="grey") # end of Aug

text(31+28+31+30+31+30+31+31+15, 29, "Sep")

abline(v= 31+28+31+30+31+30+31+31+30, col="grey") # end of Sept

text(31+28+31+30+31+30+31+31+30+15, 29, "Oct")

abline(v= 31+28+31+30+31+30+31+31+30+31, col="grey") # end of Oct

summary(dframe1$Species)

points(No ~ J.day, data = dframe1, subset= dframe1$Species=="Bec",

pch=19, col=rgb(0.5,0.5,0.5,0.5))

pdat<-expand.grid(J.day=seq(71,305,0.1), Species="Bec")

pdat

pred<-predict(model1,newdata=pdat,na.rm=T,type="response",se.fit=T)

pred

predframe<-data.frame(pdat,preds=pred$fit,se=pred$se.fit)

predframe

lines(predframe$preds ~ predframe$J.day, col="black", lwd =2)

lines(predframe$preds + predframe$se ~ predframe$J.day, col="black", lty =2)

lines(predframe$preds - predframe$se ~ predframe$J.day, col="black", lty =2)

#.####

#...(iv) Daubenton's ####

plot(No ~ J.day, col="white",

data = dframe1,

ylim=c(0,300),

xlim=c(50,300),

ylab = "No. of files",

xlab = "Julian day",

las = 1,

main="(iv) Daubenton's")

# Add monthly lines

abline(v = 31, col="grey") # End of Jan

abline(v= 31+28, col="grey") # end of Feb

text(31+28+15, 290, "Mar")

abline(v= 31+28+31, col="grey") # end of Mar

text(31+28+31+15, 290, "Apr")

abline(v= 31+28+31+30, col="grey") # end of April

text(31+28+31+30+15, 290, "May")

abline(v= 31+28+31+30+31, col="grey") # end of May

text(31+28+31+30+31+15, 290, "Jun")

abline(v= 31+28+31+30+31+30, col="grey") # end of June

text(31+28+31+30+31+30+15, 290, "Jul")

abline(v= 31+28+31+30+31+30+31, col="grey") # end of July

text(31+28+31+30+31+30+31+15, 290, "Aug")

abline(v= 31+28+31+30+31+30+31+31, col="grey") # end of Aug

text(31+28+31+30+31+30+31+31+15, 290, "Sep")

abline(v= 31+28+31+30+31+30+31+31+30, col="grey") # end of Sept

text(31+28+31+30+31+30+31+31+30+15, 290, "Oct")

abline(v= 31+28+31+30+31+30+31+31+30+31, col="grey") # end of Oct

summary(dframe1$Species)

points(No ~ J.day, data = dframe1, subset= dframe1$Species=="dau",

pch=19, col=rgb(0.5,0.5,0.5,0.5))

pdat<-expand.grid(J.day=seq(71,305,0.1), Species="dau")

pdat

pred<-predict(model1,newdata=pdat,na.rm=T,type="response",se.fit=T)

pred

predframe<-data.frame(pdat,preds=pred$fit,se=pred$se.fit)

predframe

lines(predframe$preds ~ predframe$J.day, col="black", lwd =2)

lines(predframe$preds + predframe$se ~ predframe$J.day, col="black", lty =2)

lines(predframe$preds - predframe$se ~ predframe$J.day, col="black", lty =2)

#.####

#...(v) Natterer's ####

plot(No ~ J.day, col="white",

data = dframe1,

ylim=c(0,2000),

xlim=c(50,300),

ylab = "No. of files",

xlab = "Julian day",

las = 1,

main="(v) Natterer's")

# Add monthly lines

abline(v = 31, col="grey") # End of Jan

abline(v= 31+28, col="grey") # end of Feb

text(31+28+15, 1900, "Mar")

abline(v= 31+28+31, col="grey") # end of Mar

text(31+28+31+15, 1900, "Apr")

abline(v= 31+28+31+30, col="grey") # end of April

text(31+28+31+30+15, 1900, "May")

abline(v= 31+28+31+30+31, col="grey") # end of May

text(31+28+31+30+31+15, 1900, "Jun")

abline(v= 31+28+31+30+31+30, col="grey") # end of June

text(31+28+31+30+31+30+15, 1900, "Jul")

abline(v= 31+28+31+30+31+30+31, col="grey") # end of July

text(31+28+31+30+31+30+31+15, 1900, "Aug")

abline(v= 31+28+31+30+31+30+31+31, col="grey") # end of Aug

text(31+28+31+30+31+30+31+31+15, 1900, "Sep")

abline(v= 31+28+31+30+31+30+31+31+30, col="grey") # end of Sept

text(31+28+31+30+31+30+31+31+30+15, 1900, "Oct")

abline(v= 31+28+31+30+31+30+31+31+30+31, col="grey") # end of Oct

summary(dframe1$Species)

points(No ~ J.day, data = dframe1, subset= dframe1$Species=="Nat",

pch=19, col=rgb(0.5,0.5,0.5,0.5))

pdat<-expand.grid(J.day=seq(71,305,0.1), Species="Nat")

pdat

pred<-predict(model1,newdata=pdat,na.rm=T,type="response",se.fit=T)

pred

predframe<-data.frame(pdat,preds=pred$fit,se=pred$se.fit)

predframe

lines(predframe$preds ~ predframe$J.day, col="black", lwd =2)

lines(predframe$preds + predframe$se ~ predframe$J.day, col="black", lty =2)

lines(predframe$preds - predframe$se ~ predframe$J.day, col="black", lty =2)

#.####

#...(vi) Whiskered/Brandt's ####

plot(No ~ J.day, col="white",

data = dframe1,

ylim=c(0,50),

xlim=c(50,300),

ylab = "No. of files",

xlab = "Julian day",

las = 1,

main="(vi) Whiskered / Brandt's")

# Add monthly lines

abline(v = 31, col="grey") # End of Jan

abline(v= 31+28, col="grey") # end of Feb

text(31+28+15, 48, "Mar")

abline(v= 31+28+31, col="grey") # end of Mar

text(31+28+31+15, 48, "Apr")

abline(v= 31+28+31+30, col="grey") # end of April

text(31+28+31+30+15, 48, "May")

abline(v= 31+28+31+30+31, col="grey") # end of May

text(31+28+31+30+31+15, 48, "Jun")

abline(v= 31+28+31+30+31+30, col="grey") # end of June

text(31+28+31+30+31+30+15, 48, "Jul")

abline(v= 31+28+31+30+31+30+31, col="grey") # end of July

text(31+28+31+30+31+30+31+15, 48, "Aug")

abline(v= 31+28+31+30+31+30+31+31, col="grey") # end of Aug

text(31+28+31+30+31+30+31+31+15, 48, "Sep")

abline(v= 31+28+31+30+31+30+31+31+30, col="grey") # end of Sept

text(31+28+31+30+31+30+31+31+30+15, 48, "Oct")

abline(v= 31+28+31+30+31+30+31+31+30+31, col="grey") # end of Oct

summary(dframe1$Species)

points(No ~ J.day, data = dframe1, subset= dframe1$Species=="WB",

pch=19, col=rgb(0.5,0.5,0.5,0.5))

pdat<-expand.grid(J.day=seq(71,305,0.1), Species="WB")

pdat

pred<-predict(model1,newdata=pdat,na.rm=T,type="response",se.fit=T)

pred

predframe<-data.frame(pdat,preds=pred$fit,se=pred$se.fit)

predframe

lines(predframe$preds ~ predframe$J.day, col="black", lwd =2)

lines(predframe$preds + predframe$se ~ predframe$J.day, col="black", lty =2)

lines(predframe$preds - predframe$se ~ predframe$J.day, col="black", lty =2)

#.####

dev.off()

#.####

# Table 1 (sex ratios of captured bats) ####

#...2017 ####

# Bechstein's

count <- matrix (c(19,2,10.5,10.5),

nrow = 2) # defines the number of rows in the matrix

count

fisher.test(count)

# Natterer's

count <- matrix (c(39,9,24,24),

nrow = 2) # defines the number of rows in the matrix

count

fisher.test(count)

# Whiskered

count <- matrix (c(35,11,23,23),

nrow = 2) # defines the number of rows in the matrix

count

fisher.test(count)

# Brandt's

count <- matrix (c(4,3,3.5,3.5),

nrow = 2) # defines the number of rows in the matrix

count

fisher.test(count)

# Daubenton's

count <- matrix (c(33,5,19,19),

nrow = 2) # defines the number of rows in the matrix

count

fisher.test(count)

#...2018 ####

# Bechstein's

count <- matrix (c(21,5,13,13),

nrow = 2) # defines the number of rows in the matrix

count

fisher.test(count)

# Natterer's

count <- matrix (c(62,5,33.5,33.5),

nrow = 2) # defines the number of rows in the matrix

count

fisher.test(count)

# Whiskered

count <- matrix (c(17,4,10.5,10.5),

nrow = 2) # defines the number of rows in the matrix

count

fisher.test(count)

# Brandt's

count <- matrix (c(1,0,0.5,0.5),

nrow = 2) # defines the number of rows in the matrix

count

fisher.test(count)

# Daubenton's

count <- matrix (c(13,1,7,7),

nrow = 2) # defines the number of rows in the matrix

count

fisher.test(count)

#...Combined 2017-2018 ####

# Bechstein's

count <- matrix (c(40,7 ,23.5,23.5),

nrow = 2) # defines the number of rows in the matrix

count

fisher.test(count)

# Natterer's

count <- matrix (c(101,14, 57.5, 57.5),

nrow = 2) # defines the number of rows in the matrix

count

fisher.test(count)

# Whiskered

count <- matrix (c(52,15, 33.5, 33.5),

nrow = 2) # defines the number of rows in the matrix

count

fisher.test(count)

# Brandt's

count <- matrix (c(5,3, 4,4),

nrow = 2) # defines the number of rows in the matrix

count

fisher.test(count)

# Daubenton's

count <- matrix (c(46,6, 26,26),

nrow = 2) # defines the number of rows in the matrix

count

fisher.test(count)

#...Comparing sex ratio between 2017 & 2018 ####

# Bechstein's

count <- matrix (c(19,2,21,5),

nrow = 2) # defines the number of rows in the matrix

count

fisher.test(count)

# Natterer's

count <- matrix (c(39,9,62,5),

nrow = 2) # defines the number of rows in the matrix

count

fisher.test(count)

# Whiskered

count <- matrix (c(35,11,17,4),

nrow = 2) # defines the number of rows in the matrix

count

fisher.test(count)

# Brandt's

count <- matrix (c(),

nrow = 2) # defines the number of rows in the matrix

count

fisher.test(count)

# Daubenton's

count <- matrix (c(33,5,13,1),

nrow = 2) # defines the number of rows in the matrix

count

fisher.test(count)

#.####

# Fig. 2. Catches of different Myotis species in 2013-18 ####

# Read in the data

dframe1 <-read.csv("Catch records 2013-18.csv", stringsAsFactors = T)

summary(dframe1)

dframe1$J.day <- 223 + dframe1$day.count

jpeg(file = "Figure 2", quality = 100, res = 250,

width = 2500, height = 2500)

par(mfrow = c(2,2))

# Bechstein's ####

library(MASS)

library(mgcv)

model1 <- gam(M.bech ~ s(J.day, fx=FALSE, k=10),

family=negbin(theta=3),

link=log,

na.action=na.exclude,

data=dframe1)

AIC(model1)

# gam.check(model1)

summary(model1)

# Calculate the overdispersion statistic.

# Overdispersion = residual devaince / residual d.f.

overdispersion <- model1$deviance / model1$df.residual

overdispersion

summary(dframe1$J.day)

plot (M.bech ~ J.day, col=rgb(0.5,0.5,0.5,0.4),

ylim=c(0,14),

xlim = c(220,285),

pch=19, data = dframe1, las=1,

xlab="Julian day", ylab="No. of bats caught",

main = "(i) Bechstein's")

# Add monthly lines

text(31+28+31+30+31+30+31+15, 13, "Aug")

abline(v= 31+28+31+30+31+30+31+31, col="grey") # end of Aug

text(31+28+31+30+31+30+31+31+15, 13, "Sep")

abline(v= 31+28+31+30+31+30+31+31+30, col="grey") # end of Sept

text(31+28+31+30+31+30+31+31+30+10, 13, "Oct")

pdat<-expand.grid(J.day=seq(223,280))

pred<-predict(model1, newdata=pdat, na.rm=T, type="response", se.fit=T)

pred

predframe<-data.frame(pdat,preds=pred$fit,se=pred$se.fit)

lines(predframe$preds ~ predframe$J.day,

lwd=2)

lines((predframe$preds+predframe$se) ~ predframe$J.day, lty=2)

lines((predframe$preds-predframe$se) ~predframe$J.day, lty=2)

# Daubenton's ####

library(MASS)

library(mgcv)

model1 <- gam(M.dau ~ s(J.day, fx=FALSE, k=10),

family=negbin(theta=3),

link=log,

na.action=na.exclude,

data=dframe1)

AIC(model1)

# gam.check(model1)

summary(model1)

# Calculate the overdispersion statistic.

# Overdispersion = residual devaince / residual d.f.

overdispersion <- model1$deviance / model1$df.residual

overdispersion

summary(dframe1$J.day)

plot (M.dau ~ J.day, col=rgb(0.5,0.5,0.5,0.4),

ylim=c(0,14),

xlim = c(220,285),

pch=19, data = dframe1, las=1,

xlab="Julian day", ylab="No. of bats caught",

main = "(ii) Daubenton's")

# Add monthly lines

text(31+28+31+30+31+30+31+15, 13, "Aug")

abline(v= 31+28+31+30+31+30+31+31, col="grey") # end of Aug

text(31+28+31+30+31+30+31+31+15, 13, "Sep")

abline(v= 31+28+31+30+31+30+31+31+30, col="grey") # end of Sept

text(31+28+31+30+31+30+31+31+30+10, 13, "Oct")

pdat<-expand.grid(J.day=seq(223,280))

pred<-predict(model1, newdata=pdat, na.rm=T, type="response", se.fit=T)

pred

predframe<-data.frame(pdat,preds=pred$fit,se=pred$se.fit)

lines(predframe$preds ~ predframe$J.day,

lwd=2)

lines((predframe$preds+predframe$se) ~ predframe$J.day, lty=2)

lines((predframe$preds-predframe$se) ~predframe$J.day, lty=2)

# Natterer's ####

library(MASS)

library(mgcv)

model1 <- gam(M.natt ~ s(J.day, fx=FALSE, k=10),

family=negbin(theta=2),

link=log,

na.action=na.exclude,

data=dframe1)

AIC(model1)

# gam.check(model1)

summary(model1)

# Calculate the overdispersion statistic.

# Overdispersion = residual devaince / residual d.f.

overdispersion <- model1$deviance / model1$df.residual

overdispersion

summary(dframe1$J.day)

plot (M.natt ~ J.day, col=rgb(0.5,0.5,0.5,0.4),

ylim=c(0,20),

xlim = c(220,285),

pch=19, data = dframe1, las=1,

xlab="Julian day", ylab="No. of bats caught",

main = "(iii) Natterer's")

# Add monthly lines

text(31+28+31+30+31+30+31+15, 18, "Aug")

abline(v= 31+28+31+30+31+30+31+31, col="grey") # end of Aug

text(31+28+31+30+31+30+31+31+15, 18, "Sep")

abline(v= 31+28+31+30+31+30+31+31+30, col="grey") # end of Sept

text(31+28+31+30+31+30+31+31+30+10, 18, "Oct")

pdat<-expand.grid(J.day=seq(223,280))

pred<-predict(model1, newdata=pdat, na.rm=T, type="response", se.fit=T)

pred

predframe<-data.frame(pdat,preds=pred$fit,se=pred$se.fit)

lines(predframe$preds ~ predframe$J.day,

lwd=2)

lines((predframe$preds+predframe$se) ~ predframe$J.day, lty=2)

lines((predframe$preds-predframe$se) ~predframe$J.day, lty=2)

# Brandt's / whiskered ####

summary(dframe1)

library(MASS)

library(mgcv)

model1 <- gam(mys.bra ~ s(J.day, fx=FALSE, k=10),

family=negbin(theta=2),

link=log,

na.action=na.exclude,

data=dframe1)

AIC(model1)

# gam.check(model1)

summary(model1)

# Calculate the overdispersion statistic.

# Overdispersion = residual devaince / residual d.f.

overdispersion <- model1$deviance / model1$df.residual

overdispersion

summary(dframe1$J.day)

plot (mys.bra ~ J.day, col=rgb(0.5,0.5,0.5,0.4),

ylim=c(0,25),

xlim = c(220,285),

pch=19, data = dframe1, las=1,

xlab="Julian day", ylab="No. of bats caught",

main = "(iv) Whiskered / Brandt's")

# Add monthly lines

text(31+28+31+30+31+30+31+15, 24, "Aug")

abline(v= 31+28+31+30+31+30+31+31, col="grey") # end of Aug

text(31+28+31+30+31+30+31+31+15, 24, "Sep")

abline(v= 31+28+31+30+31+30+31+31+30, col="grey") # end of Sept

text(31+28+31+30+31+30+31+31+30+10, 24, "Oct")

pdat<-expand.grid(J.day=seq(223,280))

pred<-predict(model1, newdata=pdat, na.rm=T, type="response", se.fit=T)

pred

predframe<-data.frame(pdat,preds=pred$fit,se=pred$se.fit)

lines(predframe$preds ~ predframe$J.day,

lwd=2)

lines((predframe$preds+predframe$se) ~ predframe$J.day, lty=2)

lines((predframe$preds-predframe$se) ~predframe$J.day, lty=2)

dev.off()

#.####

# Fig. 5. Hourly variation 2018 ####

dframe1 <- read.csv("Hourly Dusk 2018.csv")

summary(dframe1)

library(MASS)

library(mgcv)

# Treat Week as a factor

dframe1$Week<-as.factor(dframe1$Week)

#### Daubenton's ####

#set the reference week (different per species, 7 for Natt, 5 for M dau)

dframe1$Week<-relevel(dframe1$Week,ref="5")

model1 <- gam(Mdau ~ s(hr.afterdusk,fx=F, k=15, by=Week)

+ Week,

na.action=na.exclude,

data=dframe1,

family=negbin(theta=1<100, link=log))

summary(model1)

# gam.check(model1)

# Calculate the overdispersion statistic.

# Overdispersion = residual devaince / residual d.f.

overdispersion <- model1$deviance / model1$df.residual

overdispersion # 0.9737417

jpeg(file = "Figure 5", quality = 100, res = 250,

width = 2000, height = 2500)

par(mfrow = c(2,1))

plot(Mdau ~ hr.afterdusk, data=dframe1,

main="(i) Daubenton's",

ylim=c(0,60),

xlab="Hours after sunset",

ylab="No. of files",

xlim=c(0,14),

xaxt="n",

col="white")

axis(1,at= c(0,1,2,3,4,5,6,7,8,9,10,11,12,13,14), tick=TRUE,lty="solid",lwd=1.5,las=1)

abline(v=(0),lty=2,lwd=1)

text(0,30,"Sunset",srt=90,cex=1.2) # adjust formatting 200 for Nat, 30 for Dau

#Enter dusk time for each week

abline (v=(9.1),col="blue",lwd=1)

text(9.1,30,"28 Jul- 4 Aug",srt=90,cex=1.0,col="blue")

abline (v=(9.5),col="darkgreen",lwd=1)

text(9.5,30,"4 Aug - 11 Aug",srt=90,cex=1.0,col="darkgreen")

abline (v=(9.9),col="red",lwd=1)

text(9.9,30,"11 Aug -18 Aug",srt=90,cex=1.0,col="red")

abline (v=(10.32),col="orchid2",lwd=1)

text(10.32,30,"18 Aug -25 Aug",srt=90,cex=1.0,col="orchid2")

abline (v=(10.75),col="grey",lwd=1)

text(10.75,30,"25 Aug - 1 Sept",srt=90,cex=1.0,col="grey")

abline (v=(11.2),col="black",lwd=1)

text(11.2,30,"1 Sept - 8 Sept",srt=90,cex=1.0,col="black")

abline (v=(11.67),col="blue",lwd=1,lty=2)

text(11.67,30,"8 Sept - 15 Sept",srt=90,cex=1.0,col="blue")

abline (v=(12.13),col="darkgreen",lwd=1,lty=2)

text(12.13,30,"15 Sept - 22 Sept",srt=90,cex=1.0,col="darkgreen")

abline (v=(12.56),col="red",lwd=1,lty=2)

text(12.56,30,"22 Sept - 29 Sept",srt=90,cex=1.0,col="red")

abline (v=(12.97),col="orchid2",lwd=1,lty=2)

text(12.97,30,"29 Sept - 06 Oct",srt=90,cex=1.0,col="orchid2")

abline (v=(13.48),col="grey",lwd=1,lty=2)

text(13.48,30,"06 Oct - 13 Oct",srt=90,cex=1.0,col="grey")

abline (v=(13.93),col="black",lwd=1,lty=2)

text(13.93,30,"13 Oct - 20 Oct",srt=90,cex=1.0,col="black")

# Week 1

pdat<-expand.grid(hr.afterdusk=seq(0,10,0.1),Week=c("1"))

pred<-predict(model1,newdata=pdat,na.rm=T,type="response",se.fit=T)

predframe<-data.frame(pdat,preds=pred$fit,se=pred$se.fit)

lines(predframe$preds[predframe$Week=='1']~predframe$hr.afterdusk[predframe$Week=='1'],

lwd=2,col="blue")

# Week 2

pdat<-expand.grid(hr.afterdusk=seq(0,10,0.1),Week=c("2"))

pred<-predict(model1,newdata=pdat,na.rm=T,type="response",se.fit=T)

predframe<-data.frame(pdat,preds=pred$fit,se=pred$se.fit)

lines(predframe$preds[predframe$Week=='2']~predframe$hr.afterdusk[predframe$Week=='2'],

lwd=2,col="darkgreen")

# Week 3

pdat<-expand.grid(hr.afterdusk=seq(0,10,0.1),Week=c("3"))

pred<-predict(model1,newdata=pdat,na.rm=T,type="response",se.fit=T)

predframe<-data.frame(pdat,preds=pred$fit,se=pred$se.fit)

lines(predframe$preds[predframe$Week=='3']~predframe$hr.afterdusk[predframe$Week=='3'],

lwd=2,col="red")

# Week 4

pdat<-expand.grid(hr.afterdusk=seq(0,11,0.1),Week=c("4"))

pred<-predict(model1,newdata=pdat,na.rm=T,type="response",se.fit=T)

predframe<-data.frame(pdat,preds=pred$fit,se=pred$se.fit)

lines(predframe$preds[predframe$Week=='4']~predframe$hr.afterdusk[predframe$Week=='4'],

lwd=2,col="orchid2")

# Week 5

pdat<-expand.grid(hr.afterdusk=seq(0,11,0.1),Week=c("5"))

pred<-predict(model1,newdata=pdat,na.rm=T,type="response",se.fit=T)

predframe<-data.frame(pdat,preds=pred$fit,se=pred$se.fit)

lines(predframe$preds[predframe$Week=='5']~predframe$hr.afterdusk[predframe$Week=='5'],

lwd=2,col="grey")

# Week 6

pdat<-expand.grid(hr.afterdusk=seq(0,12,0.1),Week=c("6"))

pred<-predict(model1,newdata=pdat,na.rm=T,type="response",se.fit=T)

predframe<-data.frame(pdat,preds=pred$fit,se=pred$se.fit)

lines(predframe$preds[predframe$Week=='6']~predframe$hr.afterdusk[predframe$Week=='6'],lty=1,

lwd=1,col="black")

# Week 7

pdat<-expand.grid(hr.afterdusk=seq(0,12,0.1),Week=c("7"))

pred<-predict(model1,newdata=pdat,na.rm=T,type="response",se.fit=T)

predframe<-data.frame(pdat,preds=pred$fit,se=pred$se.fit)

lines(predframe$preds[predframe$Week=='7']~predframe$hr.afterdusk[predframe$Week=='7'],lty=2,

lwd=2,col="blue")

# Week 8

pdat<-expand.grid(hr.afterdusk=seq(0,12,0.1),Week=c("8"))

pred<-predict(model1,newdata=pdat,na.rm=T,type="response",se.fit=T)

predframe<-data.frame(pdat,preds=pred$fit,se=pred$se.fit)

lines(predframe$preds[predframe$Week=='8']~predframe$hr.afterdusk[predframe$Week=='8'],lty=2,

lwd=2,col="darkgreen")

# Week 9

pdat<-expand.grid(hr.afterdusk=seq(0,13,0.1),Week=c("9"))

pred<-predict(model1,newdata=pdat,na.rm=T,type="response",se.fit=T)

predframe<-data.frame(pdat,preds=pred$fit,se=pred$se.fit)

lines(predframe$preds[predframe$Week=='9']~predframe$hr.afterdusk[predframe$Week=='9'],lty=2,

lwd=2,col="red")

# Week 10

pdat<-expand.grid(hr.afterdusk=seq(0,13,0.1),Week=c("10"))

pred<-predict(model1,newdata=pdat,na.rm=T,type="response",se.fit=T)

predframe<-data.frame(pdat,preds=pred$fit,se=pred$se.fit)

lines(predframe$preds[predframe$Week=='10']~predframe$hr.afterdusk[predframe$Week=='10'],lty=2,

lwd=2,col="orchid2")

# Week 11

pdat<-expand.grid(hr.afterdusk=seq(0,14,0.1),Week=c("11"))

pred<-predict(model1,newdata=pdat,na.rm=T,type="response",se.fit=T)

predframe<-data.frame(pdat,preds=pred$fit,se=pred$se.fit)

lines(predframe$preds[predframe$Week=='11']~predframe$hr.afterdusk[predframe$Week=='11'],lty=2,

lwd=2,col="grey")

# Week 12

pdat<-expand.grid(hr.afterdusk=seq(0,14,0.1),Week=c("12"))

pred<-predict(model1,newdata=pdat,na.rm=T,type="response",se.fit=T)

predframe<-data.frame(pdat,preds=pred$fit,se=pred$se.fit)

lines(predframe$preds[predframe$Week=='12']~predframe$hr.afterdusk[predframe$Week=='12'],lty=2,

lwd=2,col="black")

#### Natterer's ####

#set the reference week (different per species, 7 for Natt, 5 for M dau)

dframe1$Week<-relevel(dframe1$Week,ref="7")

model1 <- gam(Mnat ~ s(hr.afterdusk,fx=F, k=15, by=Week)

+ Week,

na.action=na.exclude,

data=dframe1,

family=negbin(theta=1<100, link=log)) # theta = 1.2

summary(model1)

# gam.check(model1)

# Calculate the overdispersion statistic.

# Overdispersion = residual devaince / residual d.f.

overdispersion <- model1$deviance / model1$df.residual

overdispersion # 1.248417

plot(Mnat ~ hr.afterdusk, data=dframe1,

main="(ii) Natterer's",

ylim=c(0,300),

xlab="Hours after sunset",

ylab="No. of files",

xlim=c(0,14),

xaxt="n",

col="white")

axis(1,at= c(0,1,2,3,4,5,6,7,8,9,10,11,12,13,14), tick=TRUE,lty="solid",lwd=1.5,las=1)

abline(v=(0),lty=2,lwd=1)

text(0,200,"Sunset",srt=90,cex=1.2) # adjust formatting 200 for Nat, 30 for Dau

#Enter dusk time for each week

abline (v=(9.1),col="blue",lwd=1)

text(9.1,200,"28 Jul- 4 Aug",srt=90,cex=1.0,col="blue")

abline (v=(9.5),col="darkgreen",lwd=1)

text(9.5,200,"4 Aug - 11 Aug",srt=90,cex=1.0,col="darkgreen")

abline (v=(9.9),col="red",lwd=1)

text(9.9,200,"11 Aug -18 Aug",srt=90,cex=1.0,col="red")

abline (v=(10.32),col="orchid2",lwd=1)

text(10.32,200,"18 Aug -25 Aug",srt=90,cex=1.0,col="orchid2")

abline (v=(10.75),col="grey",lwd=1)

text(10.75,200,"25 Aug - 1 Sept",srt=90,cex=1.0,col="grey")

abline (v=(11.2),col="black",lwd=1)

text(11.2,200,"1 Sept - 8 Sept",srt=90,cex=1.0,col="black")

abline (v=(11.67),col="blue",lwd=1,lty=2)

text(11.67,200,"8 Sept - 15 Sept",srt=90,cex=1.0,col="blue")

abline (v=(12.13),col="darkgreen",lwd=1,lty=2)

text(12.13,200,"15 Sept - 22 Sept",srt=90,cex=1.0,col="darkgreen")

abline (v=(12.56),col="red",lwd=1,lty=2)

text(12.56,200,"22 Sept - 29 Sept",srt=90,cex=1.0,col="red")

abline (v=(12.97),col="orchid2",lwd=1,lty=2)

text(12.97,200,"29 Sept - 06 Oct",srt=90,cex=1.0,col="orchid2")

abline (v=(13.48),col="grey",lwd=1,lty=2)

text(13.48,200,"06 Oct - 13 Oct",srt=90,cex=1.0,col="grey")

abline (v=(13.93),col="black",lwd=1,lty=2)

text(13.93,200,"13 Oct - 20 Oct",srt=90,cex=1.0,col="black")

# Week 1

pdat<-expand.grid(hr.afterdusk=seq(0,10,0.1),Week=c("1"))

pred<-predict(model1,newdata=pdat,na.rm=T,type="response",se.fit=T)

predframe<-data.frame(pdat,preds=pred$fit,se=pred$se.fit)

lines(predframe$preds[predframe$Week=='1']~predframe$hr.afterdusk[predframe$Week=='1'],

lwd=2,col="blue")

# Week 2

pdat<-expand.grid(hr.afterdusk=seq(0,10,0.1),Week=c("2"))

pred<-predict(model1,newdata=pdat,na.rm=T,type="response",se.fit=T)

predframe<-data.frame(pdat,preds=pred$fit,se=pred$se.fit)

lines(predframe$preds[predframe$Week=='2']~predframe$hr.afterdusk[predframe$Week=='2'],

lwd=2,col="darkgreen")

# Week 3

pdat<-expand.grid(hr.afterdusk=seq(0,10,0.1),Week=c("3"))

pred<-predict(model1,newdata=pdat,na.rm=T,type="response",se.fit=T)

predframe<-data.frame(pdat,preds=pred$fit,se=pred$se.fit)

lines(predframe$preds[predframe$Week=='3']~predframe$hr.afterdusk[predframe$Week=='3'],

lwd=2,col="red")

# Week 4

pdat<-expand.grid(hr.afterdusk=seq(0,11,0.1),Week=c("4"))

pred<-predict(model1,newdata=pdat,na.rm=T,type="response",se.fit=T)

predframe<-data.frame(pdat,preds=pred$fit,se=pred$se.fit)

lines(predframe$preds[predframe$Week=='4']~predframe$hr.afterdusk[predframe$Week=='4'],

lwd=2,col="orchid2")

# Week 5

pdat<-expand.grid(hr.afterdusk=seq(0,11,0.1),Week=c("5"))

pred<-predict(model1,newdata=pdat,na.rm=T,type="response",se.fit=T)

predframe<-data.frame(pdat,preds=pred$fit,se=pred$se.fit)

lines(predframe$preds[predframe$Week=='5']~predframe$hr.afterdusk[predframe$Week=='5'],

lwd=2,col="grey")

# Week 6

pdat<-expand.grid(hr.afterdusk=seq(0,12,0.1),Week=c("6"))

pred<-predict(model1,newdata=pdat,na.rm=T,type="response",se.fit=T)

predframe<-data.frame(pdat,preds=pred$fit,se=pred$se.fit)

lines(predframe$preds[predframe$Week=='6']~predframe$hr.afterdusk[predframe$Week=='6'],lty=1,

lwd=1,col="black")

# Week 7

pdat<-expand.grid(hr.afterdusk=seq(0,12,0.1),Week=c("7"))

pred<-predict(model1,newdata=pdat,na.rm=T,type="response",se.fit=T)

predframe<-data.frame(pdat,preds=pred$fit,se=pred$se.fit)

lines(predframe$preds[predframe$Week=='7']~predframe$hr.afterdusk[predframe$Week=='7'],lty=2,

lwd=2,col="blue")

# Week 8

pdat<-expand.grid(hr.afterdusk=seq(0,12,0.1),Week=c("8"))

pred<-predict(model1,newdata=pdat,na.rm=T,type="response",se.fit=T)

predframe<-data.frame(pdat,preds=pred$fit,se=pred$se.fit)

lines(predframe$preds[predframe$Week=='8']~predframe$hr.afterdusk[predframe$Week=='8'],lty=2,

lwd=2,col="darkgreen")

# Week 9

pdat<-expand.grid(hr.afterdusk=seq(0,13,0.1),Week=c("9"))

pred<-predict(model1,newdata=pdat,na.rm=T,type="response",se.fit=T)

predframe<-data.frame(pdat,preds=pred$fit,se=pred$se.fit)

lines(predframe$preds[predframe$Week=='9']~predframe$hr.afterdusk[predframe$Week=='9'],lty=2,

lwd=2,col="red")

# Week 10

pdat<-expand.grid(hr.afterdusk=seq(0,13,0.1),Week=c("10"))

pred<-predict(model1,newdata=pdat,na.rm=T,type="response",se.fit=T)

predframe<-data.frame(pdat,preds=pred$fit,se=pred$se.fit)

lines(predframe$preds[predframe$Week=='10']~predframe$hr.afterdusk[predframe$Week=='10'],lty=2,

lwd=2,col="orchid2")

# Week 11

pdat<-expand.grid(hr.afterdusk=seq(0,14,0.1),Week=c("11"))

pred<-predict(model1,newdata=pdat,na.rm=T,type="response",se.fit=T)

predframe<-data.frame(pdat,preds=pred$fit,se=pred$se.fit)

lines(predframe$preds[predframe$Week=='11']~predframe$hr.afterdusk[predframe$Week=='11'],lty=2,

lwd=2,col="grey")

# Week 12

pdat<-expand.grid(hr.afterdusk=seq(0,14,0.1),Week=c("12"))

pred<-predict(model1,newdata=pdat,na.rm=T,type="response",se.fit=T)

predframe<-data.frame(pdat,preds=pred$fit,se=pred$se.fit)

lines(predframe$preds[predframe$Week=='12']~predframe$hr.afterdusk[predframe$Week=='12'],lty=2,

lwd=2,col="black")

dev.off()
